# Varroa mite resistance in a hybrid honey bee (*Apis mellifera)* population in Southern California

**DOI:** 10.1038/s41598-026-45759-9

**Published:** 2026-03-27

**Authors:** Genesis Chong-Echavez, Boris Baer

**Affiliations:** Department of Entomology, Center for Integrative Bee Research (CIBER), University of California, Riverside, CA USA

**Keywords:** Host-parasite interactions, Honey bee health, *Varroa destructor*, Mite-resistant honey bees, Hybrid population, Ecological adaptation, Ecology, Ecology, Zoology

## Abstract

Honey bees (*Apis mellifera*) are important ecological and agricultural pollinators. In the United States, beekeepers experience substantial annual colony losses, largely driven by parasites such as the mite *Varroa destructor*. We studied a Californian hybrid honey bee population in Southern California, a genetic mix of Western European, Eastern European, Middle Eastern, and African lineages. We predicted that these bees would show lower mite infestation levels because they survive and persist without human intervention. To test this, we monitored 236 colonies over a four-year period. We found that Californian hybrid honey bee colonies consistently had lower mite infestation rates compared to colonies headed by queens from a commercial stock. Consequently, they exceeded standard treatment thresholds (≥ 3 mites per 100 worker bees) less frequently and therefore received fewer miticide treatments. We then conducted laboratory-based-choice assays to test whether colony-level differences were reflected at the brood level. Mites were significantly less attracted to seven-day-old larvae of the Californian hybrid genotype compared to commercial larvae, indicating reduced brood attractiveness. Together, our findings indicate that this Californian hybrid population experiences lower Varroa burdens under field conditions and exhibits reduced brood attractiveness to mites under controlled laboratory conditions. This population represents a valuable resource for investigating ecological, genetic, and behavioral mechanisms underlying host resistance.

## Introduction

Pollination services provided by insects are essential for ecosystem stability and food production, but have been taken for granted in the past^1–3^. Over the past two decades, substantial declines in pollinator populations have been documented^4–6^ although the global number of managed honey bee (*Apis mellifera*) colonies has generally increased over recent decades^7^. Nevertheless, beekeepers in the United States continue to experience unsustainably high annual colony losses, with reported losses of 40–50% in recent years^8,9^ and up to 62% in 2025^10^. Multiple factors have been linked to these losses, including pesticide exposure^11^, habitat loss^12^, climate change^13–15^, nutritional stress^12,16^, and exposure to parasites and pathogens, often acting synergistically^17,18^. Among these stressors, parasites and pathogens are consistently identified as the most significant drivers of colony failure^19,20^. Honey bees host an unusually high diversity of parasites and pathogens compared to other social insects^21^, and several are highly virulent, causing substantial mortality at both the individual and colony levels^22^. Furthermore, the widespread transportation of colonies across large geographic areas has exposed honey bees to novel parasites^23,24^. Such recent host range expansions and introductions are well documented, for example, for the small hive beetle (*Aethina tumida*)^25,26^, the fungal pathogen *Nosema ceranae*^27–29^, and several species of *Tropilaelaps* mites^30–32^.

Among these invasive and expanding parasites, the most consequential is the mite *Varroa destructor*^33,34^, which European honey bees acquired from Eastern honey bees (*Apis cerana*)^35^ and was reported for the first time in the 1950s^36^. In the absence of any naturally selected resistance traits in their new hosts, these mites readily propagate on developing brood, as their reproduction occurs exclusively within capped cells^37^. As mite reproduction occurs exclusively within host brood cells, their successful invasion represents a key bottleneck for mite population growth and fitness^38^. Through repeated reproduction in brood and subsequent parasitism of adult workers, infestations propagate across successive generations within the colony, progressively increasing host exposure to parasitic stress^39^. As a consequence, infested bees experience direct physiological damage when mites feed on their hemolymph or fat body tissue^40^, removing essential tissues and causing an 11–19% reduction in body weight^37,41^. This feeding damage facilitates the transmission of several honey bee viruses, including deformed wing virus (DWV), acute bee paralysis virus (ABPV), and black queen cell virus (BQCV)^42–44^, accelerating health declines. Together, these effects impair cognitive functions such as learning or navigation^45^, and reduce foraging and homing success^46^. At the colony level, mite infestations disrupt key activities such as thermoregulation^47,48^ and brood rearing, in part because reduced hypopharyngeal gland size limits nurse bees’ ability to secrete larval food (royal jelly)^49,50^. Mite infestations can also induce swarming behavior, causing colonies to abandon their brood and stored resources^51^. The global transportation of honey bees facilitated the spread of *V. destructor*, allowing these mites to infest all major *A. mellifera* populations worldwide^52^. As a result, continuous and labor-intensive mite management has become a critical component of modern beekeeping.

Host-parasite interactions can trigger rapid co-evolutionary changes in hosts because of strong natural selection for effective immune defenses^53^. Such selection can also be expected in honey bees, especially in populations that are not under human management and are not treated for diseases^54^. For example, Africanized honey bees arose following the interbreeding of the African subspecies (*Apis mellifera scutellata*) with European-derived populations. These bees escaped from a breeding program in Brazil in the 1950s, and they subsequently spread widely throughout Central and South America^55^. They successfully survive and prosper in the wild, although they have also earned a reputation for heightened defensiveness^56^. These bees are known to harbor Varroa mites and are therefore assumed to successfully manage naturally occurring mite infestations^57^. Similarly, Southern California is also home to an unmanaged honey bee population. Recent genetic analyses of these bees revealed that they are hybrids of Western European, Eastern European, and Middle Eastern honey bees^58^. They also have some African ancestry, which is substantially lower compared to the Africanized honey bees in Central and South America^58^. The Californian honey bees, therefore, represent a genetically distinct population (Allen, UC Riverside, personal communication). They are extremely widespread throughout Southern California, from San Diego to Fresno. Interestingly, a recent survey among local beekeepers revealed that over 60% have started to keep these honey bees for commercial and recreational purposes (Chong-Echavez et al., submitted).

Host responses to parasites such as *V. destructor* can be conceptualized along a spectrum from susceptibility to resistance and tolerance. A susceptible host fails to control infestation or mitigate its effects^21^. In contrast, resistance refers to traits that actively reduce parasite fitness^21^, while tolerance describes the ability to maintain health despite persistent infection^21^. In this study, we focus on resistance-associated phenotypes defined here as traits that reduced parasite load or parasite reproductive success, rather than tolerance mechanisms that mitigate damage without affecting parasite load. We examined whether a Californian hybrid honey bee population expresses such phenotypes when compared with a commercially bred European-derived stock. Accordingly, we combined long-term field monitoring conducted over a four-year period encompassing all seasons (spring, summer, autumn, and winter) with laboratory experiments to assess differences in infestation dynamics and mite host choice between genotypes. In the field, we quantified colony-level mite burden and treatment frequencies under standardized management conditions. In the laboratory, we conducted host-choice assays to test whether mites differed in their preference for brood across genotypes, allowing us to determine whether colony-level patterns are reflected in brood-level host–parasite interactions. Although *V. destructor* typically invades brood shortly before cell capping^59^, our objective was not to characterize canonical invasion timing but to test whether host genotype modifies the developmental window during which larvae become attractive to mites. If brood traits associated with resistance are expressed developmentally, differences in mite attraction between genotypes may already be detectable during earlier stages of larval development. We therefore quantified mite host choice across multiple larval age classes to test whether the timing of brood attractiveness differs across development.

## Results

### Varroa infestations in Californian hybrid and commercial honey bee colonies

**Fig. 1:**
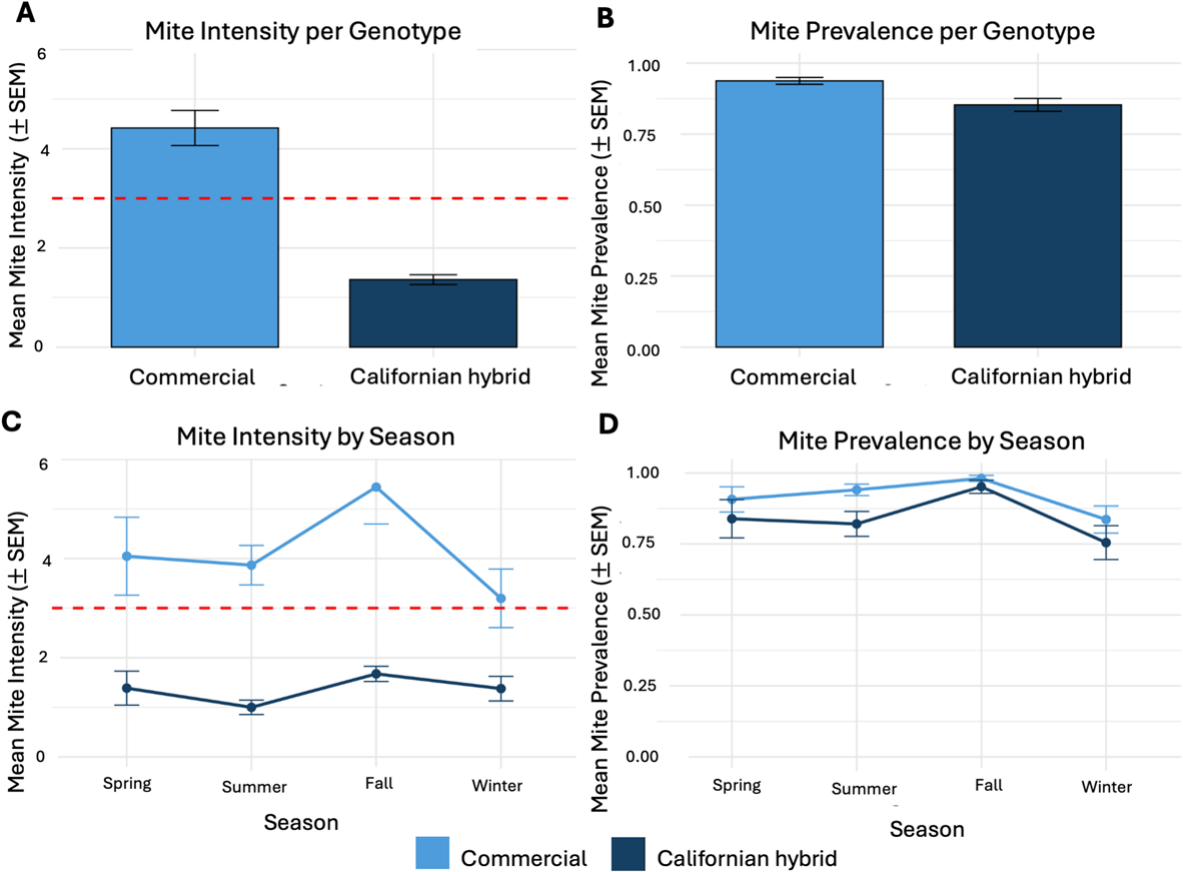
Mite intensity and prevalence in Californian hybrid and commercial honey bee colonies. (A) Mean ± SEM mite intensity per 100 adult bees by genotype. Dashed line indicates the recommended treatment threshold (3 mites per 100 bees). (B) Proportion of colonies with detectable mite infestations (mean ± SEM) by genotype. (C - D) Seasonal variation in mite intensity (C) and prevalence (D) for both genotypes. In panel C, the dashed line also denotes the recommended treatment threshold. Seasons are presented in the order Spring, Summer, Fall, and Winter, following chronological sequence of sampling across the study period.

We analyzed data from 645 colony inspections obtained from 236 unique colonies (*n* = 135 commercial; *n* = 101 Californian). Varroa *mite intensity* (defined as the number of mites per 100 adult bees) was substantially higher in commercial colonies (mean ± SEM: 4.83 ± 0.28 mites per 100 bees; *n* = 400 inspections) than in Californian hybrid colonies (1.26 ± 0.12 mites per 100 bees; *n* = 245 inspections), with Californian hybrid colonies exhibiting 68.3% lower mite intensity (Fig. 1A; Table 1). Statistical analyses confirmed a strong effect of genotype on mite intensity (Table 1). There was no seasonal effect in mite intensity detected when season was modelled as a continuous covariate (Fig. 1C; Table 1).

Mite infestation *prevalence* (proportion of inspections in which mites were detected) was high overall, with mites detected in 85.1% of inspections, but was consistently lower in Californian hybrid colonies (85.3% ± 2.3%; *n* = 191/245 inspections) than in commercial colonies (93.8% ± 1.2%; *n* = 356/400 inspections) (Fig. 1B). Statistical analyses confirmed a significant genotype effect on infestation probability (Table 1). As for mite intensity, no season pattern in prevalence was detected (Fig. 1D; Table 1).

**Table 1:** Effects of genotype and season on Varroa mite intensity and prevalence.

Predictors	Model for mite intensity and prevalence					
	Mite intensity (negative binomial)			Mite prevalence (binomial)		
	IRR	95% CI	P-Value	OR	95% CI	P-Value
Intercept	11.47	[9.37, 14.00]	**< 0.001**	8.35	[4.26, 17.28]	**< 0.001**
Genotype	0.317	[0.261, 0.385]	**< 0.001**	0.42	[0.24, 0.72]	**0.0019**
Season	1.0004	[0.900, 1.001]	0.372	1.003	[1.000, 1.006]	0.054
Random effects						
Variance	0.0699			Not included		
SD (Colony ID)	0.264			NA		
ICC	0.088			0.19		
N (Colony ID)	236			236		
Observations	645			645		
Marginal $\:{R}^{2}$/ Conditional $\:{R}^{2}$	0.288 / 0.351			0.077 / —		
AIC/BIC	4104.5/4126.9			393.9/413.8		

Generalized linear mixed models (GLMM) with genotype as a fixed effect and season included as a continuous covariate. Colony identity was included as a random intercept to account for repeated measures. Mite intensity was modelled with a negative binomial distribution and prevalence with binomial distribution. Statistically significant effects (*p* < 0.05) are shown in bold. **IRR** = incidence rate ratio; **OR** = odds ratio; **CI** = confidence interval.

### Differences in mite treatments between Californian hybrid and commercial honey bee colonies

The treatment threshold for mites was exceeded significantly more often in commercial colonies (mean ± SEM: 77.5% ± 2.1%; *n* = 187/400 inspections) than in Californian hybrid colonies (53.5% ± 3.2%; *n* = 35/245 inspections). A Fisher’s exact test confirmed that Californian hybrid colonies required fewer mite treatments compared to commercial colonies (*p* < 0.001). The odds of exceeding the treatment threshold were more than five times higher in commercial than in Californian hybrid colonies (*OR* = 5.2, 95% CI: [3.46–8.16]).

### Varroa mite host choice experiments

#### Effects of larval age on mite host choice

**Fig. 2:**
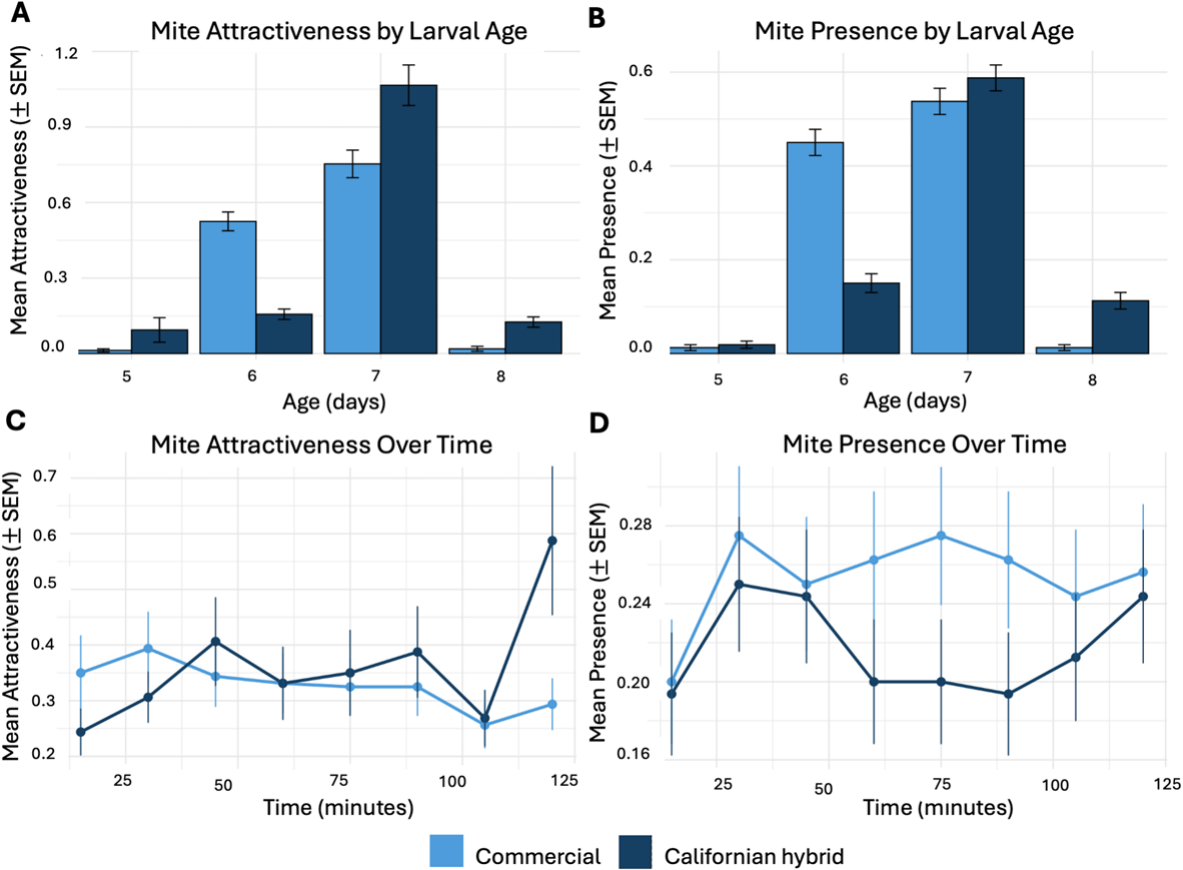
Varroa mite host choice across larval development in Californian hybrid and commercial honey bees. (A) Mean ± SEM of mite attractiveness across larval ages (five, six, seven, and eight days old) (B) Mean ± SEM of mite presence across larval ages (C - D) Mean ± SEM mite attractiveness (C) and presence (D) over the two-hour exposure time points. Points represent means across colonies (*n* = 6 per genotype, three replicates each).

We analyzed data from a total of 36 trials (*n* = 2,560 observations) using larvae aged five to eight days (*n* = 320 per age class) that originated from 12 colonies (*n* = 6 per genotype). *Mite attractiveness*, defined as the number of mites in direct physical contact with an individual larva, increased with larval age and peaked in seven-day-old larvae (mean ± SEM: 0.75 ± 0.05; *n* = 320; Fig. 2A), declining thereafter in eight-day-old larvae. Mite presence across larval age classes differed between genotypes (Table 2). Across larval ages, commercial larvae consistently showed higher mite attractiveness than Californian hybrid larvae, although the magnitude of this difference varied with age, as indicated by a significant genotype x age interaction (Fig. 2A; Table 2).

*Mite presence* was defined as the detection of at least one mite within the larval group associated with a given age class and was highest in seven-day-old larvae irrespective of genotype (Fig. 2B). Commercial larvae showed a higher probability of mite presence across all age classes (Fig. 2B; Table 2). The genotype x age interaction for mite presence was also significant (Table 2), with the difference between genotypes varying across larval ages. No effect of time since assay initiation on mite attractiveness or presence was detected across the two-hour recording period (Fig. 2C-D; Table 2). In all trials, equal numbers of 13 mites were introduced into each arena to ensure that observed differences reflect brood attractiveness rather than unequal parasite exposure.

**Table 2:** Effects of larval age and genotype on Varroa mite attractiveness and mite presence.

Predictors	Model for mite attractiveness and presence					
	Mite attractiveness (Poisson)			Mite presence (binomial)		
	IRR	95% CI	p-value	OR	95% CI	p-value
Intercept	0.12	[0.04, 0.35]	**0.001**	0.18	[0.06, 0.53]	**0.002**
Genotype	0.19	[0.04, 0.87]	**0.033**	0.06	[0.01, 0.28]	**< 0.001**
Age	1.12	[0.98, 1.27]	0.098	1.05	[0.94, 1.18]	0.400
Time	1.05	[0.96, 1.14]	0.286	1.00	[0.998, 1.004]	0.610
Genotype X Age	1.32	[1.11, 1.59]	**0.002**	1.51	[1.27, 1.80]	**< 0.001**
Random effects						
Variance	0.6853			0.7543		
SD (Colony ID)	0.8278			0.8685		
ICC	0.296			0.187		
N (Colony ID)	12			12		
Observations	2,560			2,560		
Marginal $\:{R}^{2}$/ Conditional $\:{R}^{2}$	0.046/0.328			0.033/0.214		
AIC/BIC	3667.0/3707.9			2591.1/2632.0		

Generalized linear mixed model (GLMM) with genotype, larval age, and their interaction as fixed effects and colony identity as a random intercept. Mite attractiveness was modelled using Poisson distribution and presence with a binomial distribution. Fixed effects are reported as **IRR** (incidence rate ratio) or **OR** (odds ratio) with **CI** (confidence intervals). Marginal and conditional $\:{R}^{2}$ values represent variance explained by fixed effects alone and by the full model, respectively. Statistically significant parameters (*p* < 0.05) are shown in bold.

### Effects of larval genotype on mite host choice

**Fig. 3:**
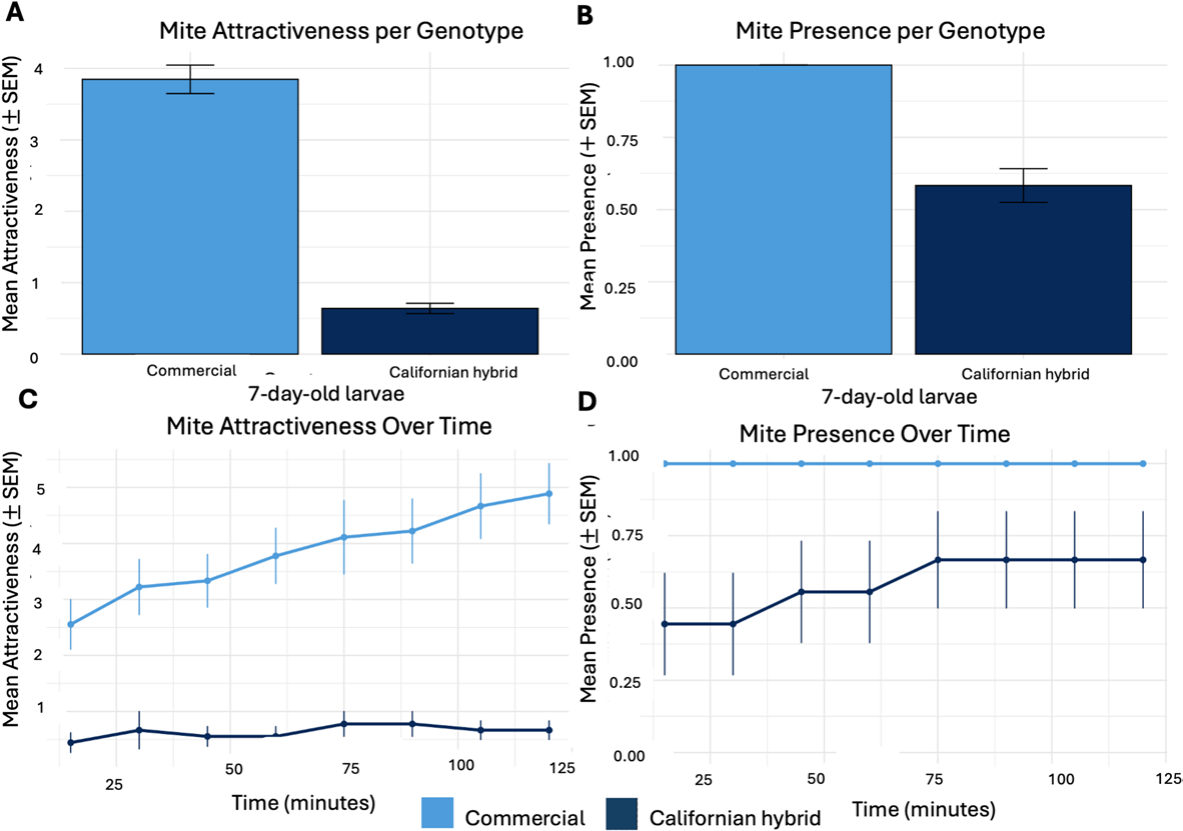
Mite host choice in seven-day-old larvae of Californian hybrid and commercial honey bees. (A) Mean ± SEM of mite attractiveness by genotype (B) Mean ± SEM of mite presence (± SEM) by genotypes. (C- D) Mite attractiveness (C) and presence (D) over the two-hour observation period. Values represent means across colonies (*n* = 3 per genotype).

Based on our finding that seven-day-old larvae exhibited the highest levels of presence, we conducted a follow-up experiment in which mites were presented with a choice between seven-day-old larvae from Californian hybrid and commercial honey bees. Larvae were sourced from six colonies (three per genotype), and a total of nine behavioral trials were conducted (*n* = 144 observations; six colonies x three replicates x eight time points). *Mite attractiveness* increased over the course of the assay, reflecting progressive host colonization during the two-hour trial (Fig. 3C).

Across the experiment, Californian hybrid larvae harbored significantly fewer mites and showed significantly lower probability of mite presence than commercial larvae (*Mite attractiveness*: mean ± SEM: 0.64 ± 0.07; *mite presence*: 58.3% ± 5.9% vs. nearly all trial in commercial; *n* = 72 larvae per genotype; Fig. 3; Table 3). Owing to near-complete imbalance in mite presence between genotypes (mites were detected on commercial larvae in nearly all trials but rarely on Californian hybrid larvae) a bias-reduced logistic regression was used to evaluate genotype effects (Table 3). In all trials, equal numbers of 10 mites were introduced into each arena to ensure that differences reflect brood attractiveness rather than unequal parasite exposure.

**Table 3:** Effects of genotype on mite attractiveness and mite presence in seven-day-old larvae.

Predictors	Model for mite attractiveness and presence					
	Mite attractiveness (Poisson)			Mite presence (Firth logistic regression)		
	*IRR*	95% CI	p-value	*OR*	95% CI	p-value
Intercept	2.67	[2.07, 3.45]	**< 0.001**	> 100	[4.32–1354]	**0.003**
Genotype	0.17	[0.10, 0.28]	**< 0.001**	0.00	[0.0006–0.156]	**0.001**
Time	1.01	[1.01, 1.02]	**0.002**	1.00	[0.996, 1.024]	0.158
Random effects						
Variance	0.0569			—		
SD (Colony ID)	0.2385			—		
ICC	0.048			—		
N (Colony ID)	6			6		
Observations	144			144		
Marginal $\:{R}^{2}$/ Conditional $\:{R}^{2}$	0.235/0.270			0.006/0.006		
AIC/BIC	612.5/630.1			102.7/117.6		

Poisson generalized linear model (GLMM) for mite attractiveness and Firth-corrected logistic regression for mite presence. Genotype was included as a fixed effect, time since assay initiation was included as covariate, and colony identity as a random intercept in the intensity model. Fixed effects are reported as **IRR** (incidence rate ratio) or **OR** (odds ratio) with **CI** (confidence intervals). Statistically significant parameters (*p* < 0.05) are shown in bold.

## Discussion

Southern California hosts a distinct and abundant population of honey bees^55,58,60^ that persists in urban and natural environments without human intervention. Local beekeepers who have collected and maintained these bees over the past decade consistently report their resilience to environmental stressors, including parasitic infestations (Chong-Echavez et al., submitted). Here, we provide multiple lines of empirical evidence showing that these bees indeed maintain lower Varroa infestation levels compared to commercial stocks. In the following sections, we place these findings within a broader ecological and evolutionary context and discuss their implications for apiculture and honey bee health.

### Varroa infestations in Californian hybrid honey bee colonies

Our four-year longitudinal analysis of colony-level data revealed that while the majority of Californian hybrid honey bee colonies were infested with *V. destructor*, they exhibited significantly lower infestation intensities compared to commercial colonies (Table 1; Fig. 1A). In addition, mite prevalence was also lower in Californian hybrid colonies (Table 1; Fig. 1B). Because the powdered sugar shake has finite sensitivity^61^, this reduced prevalence may partly reflect lower mite intensities causing mite counts to fall below the detection threshold rather than representing colonies that were indeed completely mite free. However, the consistently large difference in mite intensity between genotypes indicates that reduced prevalence is best interpreted as a downstream consequence of lower infestation levels and is therefore biologically meaningful. Californian hybrid colonies also exceeded the recommended treatment threshold significantly less often, as reported above (*p* < 0.001), supporting the interpretation this population expresses a resistance-associated phenotype.

Importantly, all colonies in this study were monitored under standardized management and were treated when mite loads exceeded intervention thresholds. Consequently, our estimates reflect resistance-associated phenotypes under managed conditions, and Varroa infestation levels in untreated colonies remain to be determined and compared in future studies. Although Californian hybrid colonies were treated less frequently because they exceeded the intervention threshold less often, our design does not allow us to fully separate the treatment effects from host genotype. Genotype categories in this study are defined by queen origin and local mating context rather than genomic assignment. Because mating occurs within a long-established admixed regional population, variation in ancestry proportion within categories is expected. Such heterogeneity would likely increase within-group variance and therefore tend to underestimate genotype-associated differences rather than inflate them. Future studies incorporating genomic ancestry estimates could further disentangle the relationship between admixture history and Varroa-associated traits.

### Climatic context and selection pressure

The climatic conditions in California are characterized by mild winters and the absence of brood-free periods and provide highly favorable conditions for Varroa population growth. Consistent with this, our colony inspections confirmed the presence of developing brood throughout the year. This continuous brood availability enables uninterrupted mite reproduction and facilitates sustained infestation pressure. This pattern contrasts sharply with temperate regions^62^, where brood production is seasonally interrupted and mite populations typically peak in summer and autumn before declining during winter and spring^63^. In line with this, we observed no significant seasonal fluctuations in mite infestation levels in either Californian hybrid or commercial colonies over the study period (Fig. 1C; Table 1). These findings indicate that honey bee colonies in Southern California experience continuous exposure to Varroa, providing a persistent ecological context in which host-parasite interactions occur throughout the year.

Such continuous brood production should impose strong and persistent natural selection for resistance traits acting at the brood level, as our laboratory assays suggest. However, despite experiencing the same climatic conditions, commercial colonies did not display similarly reduced mite burdens. One explanation is that continuous and frequent chemical treatments in managed populations reduced natural selection for host resistance traits, preventing their evolution or spread^64,65^. In contrast, unmanaged or non-treated managed populations experience continuous parasite pressure, which may allow resistance traits to increase in frequency through natural selection^54,66^.

### Varroa mite attractiveness in Californian hybrid honey bee larvae

*V. destructor* is an obligate brood parasite that requires developing honey bee larvae to reproduce^37^. Consequently, mite preference for specific larval stages is a critical determinant of mite fitness and population growth^67,68^. In our assays, mite attractiveness and presence varied with larval age (Fig. 2A-B). Regardless of genotype, seven-day-old larvae attracted the highest number of mites (Table 2). This age-dependent peak in attractiveness is consistent with previous reports^37,69–71^ and confirms that both genotypes exhibit the established temporal pattern of brood attractiveness. This comparability provides a baseline against which genotype-specific differences in age-dependent attractiveness can be evaluated, as mites preferentially invade late-stage larvae shortly before cell capping to maximize reproductive success^72^.

Across assays, Californian hybrid larvae consistently harbored significantly fewer mites and showed lower probability of mite presence than commercial larvae (Fig. 3A-B; Table 3). The genotype x time interaction was significant (Table 3), with commercial larvae maintaining near-maximum mite presence throughout the entire recording period, whereas mite presence on Californian hybrid larvae increased gradually before stabilizing at a substantially lower level (Fig. 3C-D). Notably, genotype-dependent differences were not uniform across larval ages (Table 2), indicating that the profile of age-specific brood attractiveness differed between genotypes rather than reflecting a uniform reduction across all ages. Because adult workers were excluded, these results reflect intrinsic differences in brood attractiveness under controlled laboratory conditions.

Laboratory choice assays necessarily simplify natural colony dynamics, and mites will invade brood cells even when hosts are not optimal. We therefore interpret these findings as evidence of relative differences in brood attractiveness rather than strict avoidance behavior. In genetically heterogeneous colonies composed of multiple patrilines, mites may encounter larvae expressing different genetic backgrounds and associated cues. Even modest shifts in brood attractiveness or invasion timing could influence mite reproductive success, and when occurring over multiple brood cycles significantly affect colony-level mite population growth. Whether such brood-intrinsic differences extend to mite reproductive success remains to be investigated; however, brood traits independent of adult bee behavior have been shown to directly suppress mite reproduction in naturally Varroa-resistant populations^73^, raising the possibility that similar mechanisms may contribute to the resistance observed in Californian hybrid honey bees.

Our results warrant further investigation into the molecular and chemical traits that mediate brood attractiveness. One possibility is that Californian hybrid larvae differ in volatile emissions or cuticular hydrocarbon (CHC) profiles that influence mite orientation or attachment. Age-dependent changes in brood-derived volatiles and CHCs are well documented in honey bees and have been linked to Varroa mite attraction to brood^74–76^. Differences in brood-derived chemical cues have also been implicated in the lower attractiveness of *A. cerana*, the natural host of Varroa, to invading mites^77^, with distinct larval cuticular hydrocarbons profiles shown to mediate mite cell invasion preference between the two species^72,78^, supporting the hypothesis that chemically mediated variation in brood attractiveness can translate into meaningful differences in host-parasite dynamics.

If immune-related mechanisms are involved, altered larval physiological responses to mite contact or feeding could also contribute to differences in brood-mite interactions. Previous studies have shown that *V. destructor* exposure elicits caste-and-species immune responses in developing honey bee larvae, including changes in antimicrobial peptide production and oxidative stress pathways^72^. Comparative transcriptomic or proteomic analyses between genotypes could help identify molecular pathways associated with differences in brood attractiveness or host-mite interactions. Identifying the mechanistic basis of these traits would advance our understanding of host-parasite coevolution and may inform selective breeding programs aimed at enhancing varroa resistance. Such insights would provide a stronger foundation for integrating brood-level traits into applied apiculture management strategies.

### Colony-level mechanisms

Colony level mechanisms here refer to social behaviors emerging from worker interactions (e.g., swarming, hygienic behavior, grooming), as distinct from individual/brood-level mechanism such as intrinsic larval traits that influence mite host choice. Our colony-level findings open promising avenues for future research, particularly in quantifying how differences in mite infestation rates influence colony survival, productivity, and overall fitness, especially in the absence of human-mediated mite management strategies. Further investigation is also needed to identify the specific life-history and behavioral traits that enable Californian hybrid honey bees to resist mite infestations, including the specific resistance-associated traits present in this population. Candidate mechanisms include grooming behavior^79^, Varroa-sensitive hygiene (VSH)^80^, reduced mite reproductive success^53^, shortened post-capping developmental time, and altered brood chemical profiles that interfere with mite host choice. Disentangling the relative contributions of individual-level mechanisms (e.g. brood traits) and social immunity (e.g., hygienic and grooming behaviors) will be essential for understanding how resistance emerged and is maintained in this natural populations.

Previous studies have shown that honey bees may reduce mite loads through swarming, a process in which adult workers abandoned brood and disrupt the mite reproductive cycle, analogous to a brood-free period^51,81,82^. This behavior has been documented in both European and Asian honey bee populations^83^, the latter being the original hosts of *V. destructor*. However, swarming was actively prevented in our study through routine colony splitting and queen replacement. Consequently, differences in swarming behavior cannot explain the observed variation in infestation rates. To disentangle colony-level processes from host-level resistance mechanisms, we therefore conducted laboratory assays that isolated direct brood-mite interactions.

### Varroa mite resistance in unmanaged honey bee populations

Our findings are consistent with a growing body of evidence indicating that naturally evolved resistance to *V. destructor* has independently emerged in several unmanaged *A. mellifera* populations. Notably, Africanized honey bees in South America have survived without miticidal treatments for decades, a persistence that has been linked to traits such as enhanced grooming behavior and reduced mite reproductive success^84^. Similarly, unmanaged or geographically isolated populations, such as those on the Fernando de Noronha archipelago in Brazil^85,86^, feral colonies in the Arnot Forest in the northeastern USA^87^, and treatment-free populations in Norway^66^, have also demonstrated varying degrees of naturally occurring resistance. Selectively bred European strains, including Buckfast-derived lines, exhibit strong expression of Varroa Sensitive Hygiene (VSH) and grooming behavior, further underscoring the importance of behavioral traits in mitigating Varroa infestation^68,88^.

Together, these cases support the hypothesis that sustained parasite pressure in the absence of human management can promote the evolution of mite resistance. The partial African ancestry of the Californian hybrid population raises the possibility that resistance-associated traits were introduced through introgression, followed by local adaptation in unmanaged or loosely managed environments. In contrast to Africanized honey bees, which are highly defensive, some Californian hybrid bees exhibit comparatively mild defensive behavior^89^(Watts, UC Riverside, personal communication), suggesting that resistance is not necessarily coupled to heightened aggression.

## Conclusions

We show that the Californian hybrid honey bees population maintains significantly lower *V. destructor* burdens at both the colony and brood levels relative to a commercial stock. These consistent differences under field and laboratory conditions identify this population as a valuable system for investigating factors associated with reduced mite infestation. More broadly, our findings imply that consistent parasite pressure in unmanaged honey bee populations can promote the emergence of resistance-associated phenotypes, highlighting the importance of ecological and evolutionary processes in shaping host-parasite dynamics in honey bees.

## Materials and methods

### Varroa infections affect different honey bee genotypes

#### Colony management

As part of ongoing research and teaching at the Center for Integrative Bee Research (CIBER; https://ciber.ucr.edu), we maintain around 80 honey bee colonies on the Riverside campus of the University of California, Riverside. We inspect these colonies every three weeks following standard beekeeping practices as widely used by commercial beekeepers. We mark each queen with a color dot on her thorax that allows us to track the maternal lineage of each colony. We classify colonies into two genotypes based on queen origin and mating history: (1) Colonies of the *commercial genotype are* headed by queens that we purchased from breeders in Northern California, Georgia, and Hawaii; and (2) Colonies of the *Californian hybrid genotype* were either collected from unmanaged populations or originated from colonies in which a commercial queen was naturally superseded by a locally mated queen. Because these replacement queens mated locally, their offspring represent genetically admixed individuals reflecting regional gene flow.

Colonies were classified by queen origin as a proxy for expected ancestry rather than as genetically discrete populations. In Southern California, managed and unmanaged colonies coexist within a long-established admixed population characterized by ongoing gene flow. Given that queens mate locally and are highly polyandrous, substantial within-colony and within-category genetic heterogeneity is expected. Consequently, colonies categorized as “Californian hybrid” may include both relatively recent and multi-generational admixture. This classification approach therefore captures expected differences in ancestry while acknowledging underlying genetic continuity within the regional population. Colony management practices were otherwise standardized across genotypes. Regardless of the type of queen in a colony, we provided supplemental feeding (a 1:3 sugar-to-water solution) as needed and added or removed boxes or frames as necessary, depending on the colony’s size, season, and productivity. We analyzed data that we collected from colonies over a period of four years between 2019 and 2022.

#### Quantifying Varroa mite infestations

We measured Varroa infestations using the powdered sugar shake method^90^. We collected approximately 200 adult bees from a brood frame and placed them into a jar with two teaspoons of powdered sugar. After shaking the bees for 60 s, the mites were scattered onto a white tray and then dispersed in water to visually count the mites. Bees were returned to the colony after the assay. Following standard beekeeping practices, we applied miticide treatment to any colony that exceeded the threshold of 3 mites per 100 bees^91^, regardless of genotype. We applied treatments following best-practice beekeeping standards and used formic acid in spring and thymol in fall^57^.

### Varroa mite host choice experiments

#### Larval rearing and arena setup

We reared larvae aged five to eight days in vitro from 12 honey bee colonies (*n* = 6 per genotype) following standardized methods^92^. To obtain larvae of known age, queens were confined on empty brood frames for 24 h. Larval age was defined as days post-oviposition, i.e., we counted from the day the egg was laid. This process was repeated over four consecutive days to produce larval age classes (5-, 6-, 7-, and 8-days post-oviposition). Larvae were transferred individually into pre-warmed 24-well culture plates containing 160 µl of artificial diet^93^ and incubated them at 34 °C and 60% relative humidity (RH). Live Varroa mites were collected using sugar shakes from donor colonies (see above) and maintained on moist filter paper in petri dishes at 34 °C and 60% RH until experimental use within three hours.

#### Effect of larval age on mite host choice

**Fig. 4:**
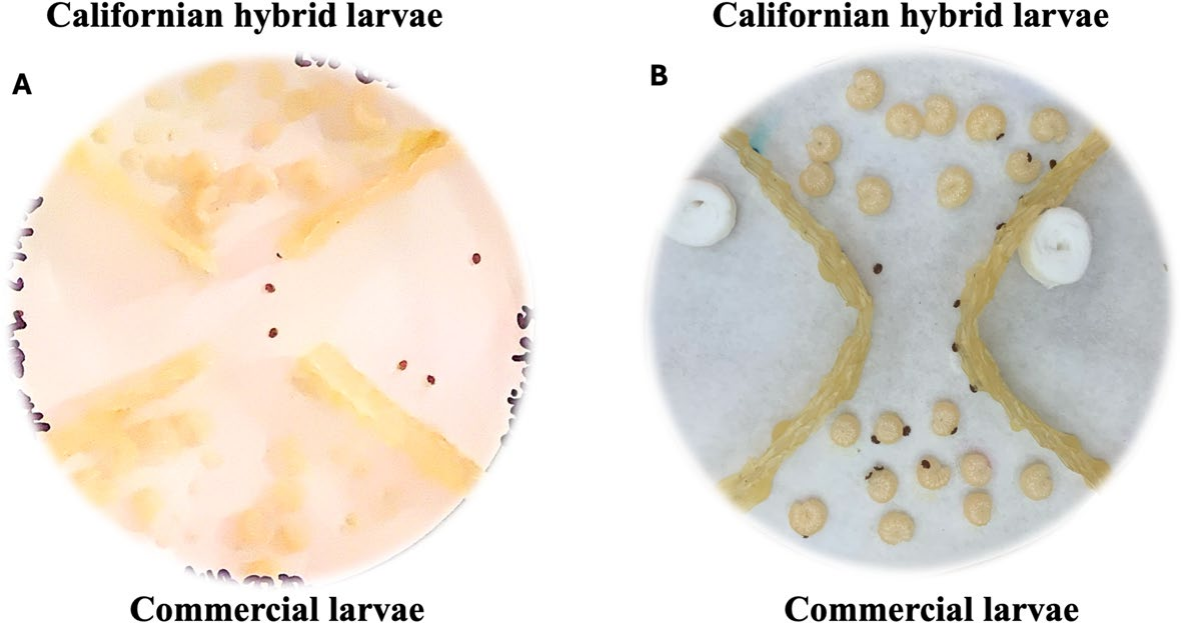
Experimental setup for Varroa mite host-choice assays using honey bee larvae of different ages and genotypes. (A) Choice arena testing for mite attractiveness to larvae of different ages (five to eight days). Beeswax strips divided the petri dishes into quadrants; larvae were placed in two quadrants, with humidified filter paper being added to the others. (B) Two-choice assay testing mite preference between genotypes. Each quadrant contained ten seven-day-old larvae of either the Californian hybrid or commercial genotype. Ten mites were released at the center, and their positions were recorded every 10 min for 120 min. All assays were conducted at 34 °C and 60% RH under red light.

To assess the effect of larval age on Varroa mite host choice, we established experimental arenas and conducted three independent trials per colonies. Each arena consisted of a 100 mm diameter Petri dish divided into four quadrants using beeswax foundation strips (Fig. 4A). Two opposing quadrants were each provisioned with 20 honey bee larvae (five individuals from each of four age groups, totaling 40 larvae per dish). The remaining two quadrants contained a humidified filter paper to prevent desiccation, as both Varroa mites and honey bee larvae are sensitive to low humidity^93,94^. Thirteen live *V. destructor* mites were released into the undivided central area of the dish, and the arenas were incubated at 34 °C under low-intensity red illumination to minimize visual disturbance. Mite positions were recorded photographically at 15-minute intervals over a total duration of 120 min.

Each larva occupied a predefined position within the arena, arranged in groups of five individuals per age class (5-, 6-, 7-, and 8-days old), with age class recorded prior to mite introduction. Mite attractiveness was quantified as the number of mites in direct physical contact with individual larva, and mite presence as the detection of at least one mite within the corresponding age class group.

#### Effect of larval genotype on mite host choice

To compare Varroa mite attraction between Californian hybrid and commercial honey bee larvae of the attractive age group, we used seven-day-old larvae from both genotypes, reared as previously described. Ten larvae of each genotype were placed in opposing quadrants of a 100 mm Petri dish (Fig. 4B), with ten mites released into the central, undivided zone. Mite positions were recorded every 10 min over 120 min under red light at 34 °C and 60% relative humidity. Larvae were obtained from six different colonies (*n* = 3 colonies per genotype), and three independent trials were conducted per colony, yielding a total of nine replicates. Fresh larvae and mites were used for each trial.

### Statistical analyses

All analyses were performed in R (version 2024.04.2 + 764) using RStudio. Figures were generated with the ggplot2 package.

### Varroa infestations in Californian hybrid and commercial honey bee colonies

Mite intensity, defined as the number of Varroa mites per 100 adult bees, was analyzed using a generalized linear mixed model (GLMM) with a negative binomial distribution and log link function to account for overdispersion in count data^95^. Genotype was included as a fixed effect, season was modeled as a continuous covariate, and Colony ID was incorporated as a random intercept to account for repeated measures within colonies. We used model coefficients to obtain *Incidence Rate Ratios* (*IRR*), which quantify the proportional change in expected mite counts to estimate the effect size of our findings. To do this, we used the raw data (i.e., mite counts per 100 bees), rather than the log-transformed data used for the GLMM. Confidence intervals (CIs) for *IRR* were calculated using the Wald method and are based on the log-transformed coefficient to generate symmetric intervals on the exponentiated scale. These were obtained using the *confint* function in the *stats* and *lme4* packages in R, using default asymptotic standard errors. Summary statistics are always reported as mean ± standard error of the mean (SEM). To avoid bias due to unequal sampling frequency among individual colonies, the mean mite intensity was first calculated per colony (Colony ID), and SEM was then computed across colonies. This ensured that each colony contributed equally to genotype-level summaries regardless of the number of inspections.

### Mite prevalence in colonies

Mite prevalence was defined as a binary outcome coded as 1 if any mites were detected (mite count > 0) and 0 otherwise. Prevalence was first analyzed at the level of individual inspections using a GLMM with a binomial distribution and logit link function, applying the same fixed and random effects structure as in the intensity model (see above). However, due to singularity in the random effect structure, the variance associated with Colony ID was estimated as zero. This outcome suggests that including Colony ID did not improve the model fit, likely because the prevalence outcome did not vary substantially within colonies beyond what was captured by the fixed effects. We then used a generalized linear model (GLM) without Colony ID as a random effect. For mite prevalence, we used model coefficients to calculate *Odds Ratios* (*OR*) to quantify the change in odds of detecting mite presence (prevalence) associated with each predictor^96^. This allowed us to compare infestation probabilities across genotypes and over time. Coefficient intervals (CIs) of OR were estimated using the Wald method as described for IFF above.

### Seasonal effects

We also tested for any effects of season on mite infestation rates, which have been documented in previous research^63^. To capture temporal variation across the year without imposing discrete seasonal categories, inspection date was included as a continuous covariate. Each inspection date was converted to a numeric day of the year (Julian day) using the *yday* function in the *lubridate* package in R. This approach allowed us to model gradual seasonal trends in mite dynamics across the annual cycle while avoiding arbitrary classification of seasons (e.g., spring, summer, etc.). Treating season as a continuous predictor reduced the risk of overfitting and improved the interpretability of temporal effects.

### Differences in Varroa mite treatments between genotypes

To compare Varroa mite treatments, we calculated the proportion of colony inspections that exceeded the established Varroa treatment threshold and compared them between genotypes using a Fisher’s exact test. To quantify the effect size, we obtained *OR* and 95% confidence intervals as described above. An *OR* > 1 indicated a higher likelihood of requiring treatment of one genotype and therefore revealed differences in colony health management between Californian hybrid and commercial honey bees.

### Varroa mite host choice experiments

#### Age preference assay

To assess differences in Varroa mite host choice, we quantified mite attractiveness (i.e., the number of mites in direct physical contact with larvae of a given age and genotypes) and mite presence (i.e., whether at least one mite observed on a given larval group), every 15 min for 120 min. We fitted a GLMM with a negative binomial distribution using the *glmer.nb* function from the *lme4* package in R. Fixed effects included genotype (Californian and commercial), larval age (five–eight days), and scaled observation time (0–120 min), along with genotype × age interaction term to test whether mite attraction varied across developmental stages depending on genotypes. Colony identity was included as a random intercept, nested within genotype to account for repeated measures and the hierarchical structure of the data (each colony belonged to only one genotype).

#### Genotype preference assays

To analyze mite presence in the host-choice assays, defined here as the presence of at least one mite within a quadrant containing larvae of a given genotype, we used a binomial GLMM with a logit link and the same fixed and random structure as in the age-related analyses using the *glmer* function. For the follow-up two-choice assay using seven-day-old larvae, we compared the relative distribution of mites between genotype groups using GLMs implemented via the *glmm*TMB and *glm* functions. Mite attractiveness defined as the number of mites in direct physical contact with larvae, was modeled with Poisson GLMM including genotype, observation time, and their interaction as fixed effects, and colony as a random intercept. Due to near-complete imbalance in mite presence data across exposure time (mites were detected on commercial larvae in nearly all time points but rarely on Californian hybrid larvae), we applied Firth-corrected logistic regression to obtain unbiased estimates. Model significance was evaluated using likelihood ratio tests and Wald statistics. We also obtained incidence rate ratios (*IRR*) and odds ratios (*OR*) with corresponding 95% confidence intervals. Summary statistics are visualized as group means ± standard error of the mean (SEM) over time and by genotype.

#### Data presentation and visualization

For all models, we reported effect sizes (*IRR*/*OR*), 95% CIs, and *p*-values derived from Wald tests or likelihood ratio tests, as appropriate. Summary statistics for visualizations are presented as mean ± SEM, with SEM calculated across independent colony-level replicates. All plots were generated using the *ggplot2* package in R.

## Data Availability

The datasets generated and/or analyzed during the current study are available from the corresponding author on reasonable request.
